# Supplementary material for: Supporting Return to Work after Breast Cancer: A Mixed Method Study
Source: Healthcare (Basel). 2023 Aug 19;11(16):2343. doi: 10.3390/healthcare11162343 (PMC10454012; doi:10.3390/healthcare11162343)
Supplement: Supplementary file 1 [file healthcare-11-02343-s001.zip › healthcare-2544892-supplementary.pdf]

Supplement S1. Outline-Guide for the Structured Interview "Return To Work After Breast Cancer"

PRELIMINARY DATA ON PHYSIOLOGICAL HISTORY:

- married (yes/no)
- number of children
- physical activity
- nutrition
- diuresis/alvus
- height
- weight
- disability (recognized by evaluation commission)

1) **HEALTH PROBLEMS**

- Family medical history, focused on:
    - Familiarity for cancer
    - Familiarity for autoimmune diseases
- Remote pathological anamnesis, focused on:
    - Autoimmune diseases
    - Trauma/accidents
    - Past functional impairments or limitations
    - Past medications
- Current health status, focused on breast cancer:
    - Histological type
    - TNM Stadiation/Grading
    - Diagnostic process (how she noticed it)
    - Treatment course (mammogram, biopsy, CT scan, MRI, neoadjuvant chemo/radiation, surgery, adjuvant chemo/radiation)
    - Chemotherapy currently administered (specify)
    - Legally recognized disability (yes/no)
- Clinical outcomes of therapies:
    - Any disabling physical problems? (yes/no)
        - Functional limitations of the range of motion for the arms
        - Muscle strength/muscle tone
        - Pain
        - Lymphedema
        - Asthenia
        - Fatigue

        - Lifestyle changes
    - Any disabling mental problems? (yes/no)
        - Cognitive impairment
        - Anxiety/depression
        - Psychological support ongoing program
        - Lifestyle changes

2) WORK AND RELATIONSHIP WITH THE MANAGEMENT

- Age of starting work
- Job history (what, where, how long)
- Current job (professional category)
- Specific tasks performed in the current job (professional activities)
- Last fitness-to-work issued by your occupational physician:
    - Has she returned to work yet?
        - If yes: any prescriptions or limitations?
        - If no: choose one among: sick leave – vacation – leave from work
- Main issues at work:
    - With colleagues
    - With managers
    - Workstations and tools
    - Personal protective equipment
    - Other (specify)
- Qualitative perception of working with sickness/after sickness:
    - How do you feel about this situation?
    - What are your fears?
    - Do you feel able to do everything like before?
    - Are you afraid of being discriminated against/discharged?
    - Do you think that going back to work will have positive consequences in your life?
    - Other (specify)

3) QUALITY OF WORK ORGANIZATION

- Please, fill in the Organizational Justice Questionnaire.

VISITA MEDICINA DEL LAVORO

Data di compilazione |___|___|___|

Cognome e nome ....
Nato il |____|____|____| a _.. Nazion.:_.....................................
Domicilio abituale .... tel.: ……………………………
Mail:____

Stato familiare: Singola □	Con partner	Senza figli □	Con figli □		
Studio: Media inferiore □	Diploma □	Laurea □			
Reddito tra 0 e 15mila □	Tra 15 e 28 mila □	Tra 28 e 55mila □	Oltre 55mila □		
Peso:	Altezza:	Residenza: città □	Paese, campagna: □		

F1.Che tipo di attività fisica svolgi più regolarmente: ………………… ………………………………………

F2. Quante volte a settimana pratichi questo esercizio fino a sudare?	0	1-2	3-4	5-7
F3. Quanto tempo dedichi ogni volta a questo esercizio?	<15’	16-30’	31-60’	>60’

Classe tumore: I □	II □	III □	IV □
Trattamento: Chir. □	Radio □	Chemio □	Adiuvante □
Ha già ripreso il lavoro? NO □	SI □	Invalidità %....................	
Problemi del sonno □	Facile stancabilità □	Dolore spalla/braccio □	Linfedema □
Difficoltà concentrazione □	Deficit memoria □		

Anamnesi Lavorativa

Mansioni svolte presso l'azienda attuale (Dati forniti dal lavoratore)

Dal	al	Mansione

Fattori di rischio — Giudizio sul grado di esposizione (1 = lieve, 2 = intensa)

1. ________________________________ |___|
2. ________________________________ |___|
3. ________________________________ |___|

Orario di lavoro: (ore/sett. effettive medie) |___|___|
Lavoro notturno (turni effettuati al mese) |___|
Pendolarismo (tempo per raggiungere il lavoro) |___|___|

Anni	Lavori precedenti: Attività	Rischi

Sintesi

Classificazione del rischio

☐ movimentazione manuale carichi
☐ amianto
☐ vibrazioni corpo intero
☐ radiazioni ottiche artificiali
☐ ultrasuoni
☐ altri rischi
☐ sovraccarico biomeccanico arti superiori
☐ silice libera cristallina
☐ vibrazioni mano braccio
☐ radiaz. UV naturali
☐ atmosfere iperbariche
☐ agenti chimici
☐ agenti biologici
☐ rumore
☐ microclima severo
☐ lavoro notturno
☐ cancerogeni e mutageni
☐ videoterminali
☐ campi elettromagnetici
☐ infrasuoni
☐ radiazioni ionizzanti

N	Problemi attivi	Data	Altri problemi	Data
1				
2				
3				
4				
5				
6				
7				
8				
9				
..				

Note:

Dichiarazioni del lavoratore:

Il sottoscritto, presa visione del contenuto di questa scheda, dichiara che quanto riportato nell'anamnesi corrisponde a quanto dichiarato in ogni suo punto e che non ha sottaciuto notizie in riferimento ad eventi patologici pregressi o in corso. Inoltre, il sottoscritto esprime consenso alla gestione e al trattamento elettronico dei propri dati personali da parte del medico del lavoro, per scopi statistici o scientifici, secondo il Codice in materia di protezione dei dati personali (DL 30/6/03 n.196).

(data) .... *(firma)* ....

	Themes	
	**Barriers**	**Facilitators**
Person-related Factors	• Physical problems (pain, fatigue)<br>“Pain and tiredness deriving from therapies do not allow me to stay far from home for too long” (Patient 1)<br>"I can't move heavy objects, especially if I have to place them on high shelves. Sometimes I drop things from my hand because my grip gives way.” (Patient 4)<br>“My arm has swollen causing me some problems with movement, but I have learnt to manage it over time. the thing that bothers me most is that someone in the office might notice the difference between my two arms.” (Patient 7)<br>“Low back pain is causing me severe pain crises. The new department to which I was assigned involves performing the activity in a standing position only.” (Patient 12)<br>“When my arm is immobile for a while, or when I wake up in the morning I often feel as if there are small needles in my shoulder and right arm. This also happens to me at work when I am sitting at my desk.” (Patient 24)<br>“My arm gets tired because I have to shift loads many times a day. Even if the loads don't weigh more than 10 kg, I have to move them several times a day.” (Patient 8)<br>“I can't stand up the whole work shift” (Patient 13)<br>• Motivational blocks<br>“I often feel tired, have a hard time concentrating. Sometimes have severe pain in my legs.” (Patient 11)<br>I think maybe I have been working too much, I already have 35 years of service, maybe I need to stop” (Patient 15)<br>“The work I was doing was too modest for my abilities, now I wish the company would offer me something better now.” (Patient 16)<br>“I don't feel completely well, I need more time to fully recover.”<br>“The work environment was full of tension, I don't feel like going back into a stressful environment.” (Patient 20)<br>• Cognitive and neuropsychological problems (reduced concentration, decreased performance, apathy)<br>“I don't want to go back to work because I'm tired and aware of the advanced stage of my disease, I'm severely depressed, and I don't feel like working.” (Patient 18)	• Surgical breast reconstruction<br>“Having my breast immediately reconstructed [Editor's note. after mastectomy] prevented damaging my female appearance and the image others have of me ” (Patient 1)<br>“I was very happy with the breast reconstruction in the same surgery, because it allowed me to go back to work and everyday life without changes in my appearance” (Patient 3)<br>“The tumor was in an early stage so I only had to do surgery from which I recovered soon also thanks to breast reconstruction.” (Patient 27)<br>• Work engagement<br>“I have always had a strong attachment to work, before the illness I had taken very few days off; now I am not very well but I would be very eager to start again.” (Patient 2)<br>“I feel good and am motivated to return to work, as long as I work “humane” hours [Editor's note: do not exceed the predetermined working hours] and I am not forced to work after hours.” (Patient 10)<br>“I really want to continue working; in fact, I have never even taken a single absence at work for my medical appointments.” (Patient 12)<br>“I am concerned about being placed in another office where it is known that the workload is greater than in other offices” (Patient 14)<br>“I have always had a great desire to work, and I want to find it again.” (Patient 24)

	“I get tired easily and have trouble concentrating, although I have already been back at work for a few weeks.” (Patient 14)<br>“I fear that my work performance has decreased.” (Patient 21)<br>“I don't want to hinder my colleagues in achieving company goals.” (Patient 23)	
Company-related factors	• Work overload<br>“I don't want to be the only one who does nothing and be a burden to colleagues and subordinates and receive pitying treatment. My fear is that when I go back to work I won't have the same performance as before.” (Patient 2)<br>“I am afraid that I cannot keep up with the work and in the coordination of the workers in my group for which I am responsible.” (Patient 5)<br>“The workload that I faced before the illness is an exaggerated workload that even before the illness I was struggling to bear, but now I really would not be willing to bear, this makes me very afraid.” (Patient 10)<br>“As a result of the deficit in my right arm, I am afraid that I will not sustain the pace of work as a waitress in the pizzeria and as a cook in the cafeteria, and also I am afraid of the late hour when my shift usually ends in the pizzeria.” (Patient 24)<br>“They say the problem is over. My place in the company is still the same, I must provide the same services as before and continuously improve productivity. But I just can't do it.”(Patient 30)<br>“I'm afraid I can't handle the heavier workloads that I managed previously.” (Patient 32)<br>• Work underload<br>“I was sidelined because of my situation (Editor's note. of fragilty). They considered me unreliable and not suitable for being involved in projects because I had no future in the company. They preferred to count on someone who could offer them greater reliability .” (Patient 9)<br>“When they learned of my illness, they took away the job I was doing before. They effectively demoted me.” (Patient 26)<br>• Environmental and ergonomic factors<br>“The trolleys I have to use at work have faulty wheels and I have even more trouble moving them.” (Patient 4)<br>“My incorrect posture at work will make my health condition worse”; “I don’t think I can stand night shifts any longer.” (Patient 24)<br>“Changing my workplace is a source of severe stress for me.” (Patient 26)	• Policies for RTW<br>“My employer is extremely helpful in promoting a gradual return to work, so that I can avoid excessive fatigue.” (Patient 7)<br>• Ergonomic and schedule adjustments<br>“My Occupational Physician has already excluded me from heavy work such as guards, inspections, heavy work and physical efficiency tests.” (Patient 15)<br>• Social support from colleagues and superiors<br>“My colleagues write to me and encourage me to keep going so I can get back to work as soon as possible and they promised me that when I get back I can count on their support.” (Patient 2)<br>After a company decision to change the department, I was assigned to a department with a boss who understands and appreciates my skills.” (Patient 4)<br>“My colleagues are willing to help me in the most onerous activities” (Patient 6)<br>“My colleagues were willing to help me and arrange shifts so that I can avoid night shifts. It is a well organized company.” (Patient 9)<br>“When I have to mobilize someone, colleagues help me. Same thing for distributing meals. (Editor's note. she was a Health Care Assistant)” (Patient 13)<br>“I have very helpful colleagues, and since my surgery I have stopped traveling.” (Patient 19)<br>“I have a convenient work schedule and my colleagues are helpful and cooperative. There’s a peaceful work atmosphere” (Patient 20)

	“I have to travel a long way to get to work. Commuting is a challenging factor for RTW” (Patient 25)<br>“Being in the office with my colleagues makes me afraid of contracting COVID-19.” (Patient 28)<br>“When the manager learned that I was sick, he arranged for my transfer to a work location closer to my home. I know I can ask to be moved closer to home because I am severely handicapped, but I have no intention of doing that. I don't want to transfer, I don't feel able to fit into a work environment that I don't know.” (Patient 31)<br>• Inadequate shifts<br>“My job involves night shifts and I am afraid that they may worsen my health condition” (Patient 9)<br>“I know that working nights could make my condition worse, but I have to continue working about two night shifts a week. I can't refuse because I am hired as a freelancer.” (Patient 27)<br>“I think I can no longer sustain night shifts.” (Patient 24)<br>“My work schedule and the need for in-person meetings may destabilize my work-life balance with regard to the ongoing treatment.” (Patient 28)<br>• Employer's request for work ability assessment<br>“When the pandemic broke out, the manager sent me to a medico-legal board to assess whether I could continue working in contact with children, and the board decided that I was unfit for work because of the risk of infection.” (Patient 3)	
Society-related factors	• Unequal access to welfare benefits<br>“Access to smart working from home was granted only to people with oncological conditions undergoing treatment with immunosuppressive drugs, thus creating a human capital management disparity between companies that provide flexible work and others.” (Patient 19)<br>“I had a fixed-term contract that expired while I was on sick leave, and it was not renewed.” (Patient 12)<br>“I have been found to be temporarily unfit for duty, with Contraindications for performing any duties involving mental or physical stress.” (Patient 17)<br>• Family conflict<br>“My fiancé left me after the end of chemotherapy.” (Patient 1)<br>“I am separated from my husband, he knows I am not well, but he always fusses when he has to send me money for our child maintenance. I can't pay the rent at home.” (Patient 4)	• Telecommuting, teleworking<br>“Teleworking allowed me not to lose job opportunities because I continued to write articles and maintain correspondence from home” (Patient 1)<br>“They tried to make me do smart working, but with kindergarten children it wasn’t successful.” (Patient 3)<br>“They made me work in smart working.” (Patient 4)<br>“Fortunately, in the bank where I work, telecommuting is a mode already used before the pandemic” (Patient 5)<br>“I have a great dedication to work, during sick leave I still helped colleagues from home” (Patient 14)<br>“My job not physically taxing because I work purely on a PC, I currently work smartworking except for one day a week when I physically go to the office.” (Patient 21)<br>“I work mostly on a PC and when I am tired I have the opportunity to work from home in smart-working.” (Patient 19)<br>“My job allows me to go to attendance only once a week” (Patient 22)

	"My partner tried to take advantage of my condition to obtain custody of the children in the separation lawsuit." (Patient 4)	"The use of telecommuting has not affected work performance so far, but I do not feel able to hold up the office hours I did before the illness." (Patient 23)<br>"Paradoxically, with the pandemic, the possibility of going to the office a few days a week allows me to be calmer. I am afraid of becoming infected with COVID-19." (Patient 28)<br>"Working almost exclusively from my computer gave me the opportunity to work from home after the surgery. It was very helpful" (Patient 29)<br>• Legal & welfare benefits for workers with cancer<br>"Mine is an officer position in the administration and commissariat, so fortunately I can perform (by Law) my duties mostly on the PC." (Patient 15)<br>• Social support from family members<br>"My partner, who is also a nurse, helps me a lot." (Patient 27)

Table S1. Extensive participants' answers from the semi-structured interview questionnaire.
